# Comment on ‘Prospective multicenter validation of a machine learning model for predicting anastomotic leakage in patients with gastric adenocarcinoma undergoing total or proximal gastrectomy’

**DOI:** 10.1097/JS9.0000000000003458

**Published:** 2025-09-04

**Authors:** Chun Wang, Yang Wang, Zhihe Zeng

**Affiliations:** Department of Anesthesiology, The Second Affiliated Hospital of Dalian Medical University, Dalian, China

*Dear Editor,*

We read with great interest the recently published prospective multicenter study evaluating a real-time machine learning model for predicting anastomotic leakage (AL) in patients with gastric adenocarcinoma undergoing proximal or total gastrectomy^[1]^. The investigators should be commended for conducting a prospective multicenter validation in an area where reliable predictive tools are still lacking. Given the serious complications associated with AL, their effort to provide an intraoperative, real-time decision-support tool is both timely and clinically relevant. Nevertheless, we would like to raise several points that warrant further clarification and discussion.

First, the incidence of AL in this study (2.54%) was relatively low compared with the rates previously reported (1.7–15%)^[2,3]^. Although encouraging, this difference may partly reflect variations in perioperative management strategies or institutional expertise at participating centers. However, such a low baseline incidence complicates the interpretation of model performance, particularly the positive predictive value, which was only 4.5%. While the authors acknowledged this limitation, its clinical implications merit further elaboration, especially considering that false-positive predictions could result in unnecessary anxiety or overtreatment.

Second, although the model achieved a high negative predictive value (NPV = 0.99), the specificity was relatively modest (0.577). This raises concerns about the balance between sensitivity and specificity in clinical practice. While a high NPV supports safely exempting patients from intensive monitoring, a considerable proportion of patients may still be incorrectly classified as “high risk” without developing AL. A prospective cost-effectiveness analysis – considering resource utilization, patient burden, and downstream testing – would be helpful in determining whether the proposed 35% reduction in monitoring is both safe and economically justifiable.

Third, while the multicenter design enhances external validity, all participating institutions were tertiary hospitals in China. It remains uncertain whether the model is applicable to other surgical practices, patient populations, or healthcare systems. Moreover, most cases in this study involved laparoscopic surgery, which may not fully reflect surgical patterns worldwide. Broader international validation would increase confidence in its generalizability.

Fourth, the definition of AL partly relied on subjective criteria (e.g., surgeon judgment or expert panel review), which could introduce inter-observer variability. Harmonization with standardized international definitions could improve reproducibility and facilitate comparisons with other predictive models.

Finally, the post hoc identification of a 0.45 risk threshold – below which no false negatives were observed – is thought-provoking but should be interpreted cautiously. Optimizing the threshold on the same dataset risks overfitting. Independent validation is essential before recommending modifications to postoperative monitoring protocols based on this cut-off.

In conclusion, this study represents an important step toward integrating machine learning-based prediction tools into perioperative management of gastric cancer. Nonetheless, further validation in diverse settings, careful assessment of the clinical consequences of misclassification, and integration with existing enhanced recovery protocols will be key to realizing its full clinical potential. We congratulate the authors on their innovative work and look forward to future research addressing these challenges. The article was written in accordance with the TITAN guidelines to maintain transparency regarding AI^[4]^.

## Data Availability

Not applicable.
